# The impact of the social environment on children's mental health in a prosperous city: an analysis with data from the city of Munich

**DOI:** 10.1186/1471-2458-10-199

**Published:** 2010-04-21

**Authors:** Laura Perna, Gabriele Bolte, Heidi Mayrhofer, Gabriele Spies, Andreas Mielck

**Affiliations:** 1 Helmholtz Zentrum München, German Research Centre for Environmental Health, Institute of Health Economics and Health Care Management, Neuherberg, Germany; 2 Bavarian Health and Food Safety Authority, Department of Environmental and Occupational Epidemiology, Munich, Germany; 3 City of Munich, Department of Health and Environment, Munich, Germany

## Abstract

**Background:**

Children with a low socioeconomic position are more affected by mental difficulties as compared to children with a higher socioeconomic position. This paper explores whether this socioeconomic pattern persists in the prosperous German city of Munich which features high quality of life and coverage of children mental health specialists that lies well above the national average and is among the highest in Europe.

**Methods:**

1,265 parents of preschool children participated in a cross-sectional health survey. They were given a self-administered questionnaire (including socioeconomic variables) and the 'Strengths and Difficulties Questionnaire (SDQ)', a well-established method to identify mental difficulties among children and adolescents. Prevalence estimates for the 'SDQ-Total Difficulties Score' were calculated, with a special focus on differences by parental (resp. household) socioeconomic position. The association between parental education, household income, single parenthood, nationality, and parental working status on one hand, and their children's mental health on the other, was explored using multivariable logistic regression models. The coverage of mental health specialists per 100,000 children aged 14 or younger in the city of Munich was also calculated.

**Results:**

In Munich, the distribution of mental health difficulties among children follows the same socioeconomic pattern as described previously at the national level, but the overall prevalence is about 30% lower. Comparing different indicators of socioeconomic position, low parental education and household income are the strongest independent variables associated with mental difficulties among children (OR = 2.7; CI = 1.6 - 4.4 and OR = 2.8; CI = 1.4 - 5.6, respectively).

**Conclusions:**

Socioeconomic differences in the prevalence of childhood mental difficulties are very stable. Even in a city such as Munich, which is characterized by high quality of life, high availability of mental health specialists, and low overall prevalence of these mental difficulties, they are about as pronounced as in Germany as a whole. It can be concluded that the effect of several characteristics of socioeconomic position 'overrules' the effect of a health promoting regional environment.

## Background

More than 25% of individuals develop one or more mental health or behavioural disorders in their entire lifetime [1]. Unipolar depressive disorders are among the 20 leading causes of burden of disease, and by the year 2030 they are expected to become the first leading cause of burden of disease in the world [2]. The worldwide prevalence of childhood and adolescent mental disorders is around 20% [3]. While up to 6% of this 20% is in need of a clinical intervention [3], only approximately 15% - 20% of children with mental health problems reach the existing child mental health services, as studies conducted in Canada [4], USA [5,6] and Great Britain [7] have shown.

Since half of all lifetime cases of mental health disorders start by age 14 [8], an early detection of child and adolescent mental disorders is of great public health relevance. A well-established method for detecting signs of mental difficulties is the 'Strengths and Difficulties Questionnaire (SDQ)', a brief behavioural screening questionnaire which assesses possible problems and strengths in the areas of emotional problems, hyperactivity, behavioural problems, peer problems, and prosocial behaviour. The SDQ is a 'rough-and ready' method [9] to identify mental health difficulties that can be administered to the parents and teachers of 4- to 16-year-olds and to young people aged around 11-16. It has a specificity of 94.6% and a sensitivity of 63.3% when it is completed by all potential informants (parents, teachers, and young people aged 11 or over) [10].

In Germany, the SDQ was given to the parents of 14,478 children and adolescents aged 3-17 years, within the framework of the 'German Health Interview and Examination Survey for Children and Adolescents (KiGGS - Study)' carried out by the Robert Koch Institute in 2003-2006. The results of the study showed that about 15% of all participating children and adolescents aged 3-17 years had signs of mental health difficulties. According to the 'Total Difficulties Score (SDQ-TDS)', which is generated by summing the scores from different subscales, 10.6% of the girls and 15.8% of the boys in the age group 3-6 years were classified 'borderline' or 'abnormal', respectively [11]. The distribution of the disorders also showed a distinct socioeconomic pattern: in the lowest socioeconomic group (assessed by a combination of household income, parental educational level, and occupational status), 24.1% of the children were classified as 'borderline' or 'abnormal', compared to 6.7% in the highest socioeconomic group [11].

The question whether there is a regional pattern - in addition to this socioeconomic pattern - and whether a high availability of mental health specialists has an impact on the socioeconomic pattern has not yet been addressed in Germany. It would be important, though, to study the impact of the presence of both of these two indicators on the socioeconomic distribution of mental health difficulties of children. On one hand, it could be hypothesized that socioeconomic differences decrease in a health-promoting regional and social environment (characterized by high levels of overall quality of life and prosperity, and high availability of mental health specialists). On the other hand, similar socioeconomic differences in different regions would indicate that these socioeconomic differences are very stable, independently of quality of life and of availability of mental health specialists. This would have important implications for the German health care system (in particular for the role of mental health specialists) and for the implementation of public health interventions.

We address this question by analysing the mental health status of children aged 5-7 years in the German city of Munich. We focused on this city as it features characteristics that make this urban setting very interesting in the German and international context. In Munich, in fact, the quality of life ranks among the highest worldwide [12], and the percentage of children who are at risk of poverty equates the percentage of European countries such as Denmark and Finland, which have the lowest rate of children at risk of poverty in the European Union (10%) [13,14]. Furthermore, the availability of children mental health specialists is in Munich well above the national average and among the highest in Europe [15].

This provides a very good environment to explore whether excellent living conditions coupled with excellent coverage of mental health specialists have an impact on the socioeconomic distribution of mental health difficulties among preschool children.

## Methods

### Sample

In 2004, 'health monitoring units (Gesundheits-Monitoring-Einheiten - GME)' were established in the southern German state of Bavaria in three rural and three urban regions, based on a cooperation between local health authorities, the Bavarian Health and Food Safety Authority and external partners. The objective of the GME is to collect relevant current data on child health in Bavaria [16]. Within the framework of the GME, a cross-sectional health survey was carried out during the 2005/2006 compulsory school entrance health examination. The SDQ, together with a self-administered questionnaire containing *inter alia *questions on socioeconomic variables, was given to the parents of the children who were attending the examination. The survey was approved by the Ethical Committee of the Bavarian Chamber of Physicians. Written informed consent was obtained from all parents answering the questionnaire. The total Bavarian sample consisted of 6,206 children (response rate 73%). In Munich, given the high total number of preschool children (9,949), a subset of 19 schools was randomly selected from 14 city districts so as to be representative of the social situation in Munich [17]. The study population comprises 1,265 children (637 girls and 628 boys, response rate 69%) with a median age of 5.9 years. Under these children 1,172 had a completed SDQ (589 girls and 583 boys).

From the Munich health authority we obtained the list of corresponding medical specialists (child and adolescent psychiatrists, child and adolescent psychologists, medical doctors specialised in psychosomatics and psychotherapy) practicing in Munich as of 2008. The other demographic data relating to the city of Munich that we used (age distribution of the population) dates to 2007 and is freely available on the website of the city of Munich [18].

### Socioeconomic indicators

Several questions in the questionnaire were posed in order to identify the social environment in which the children live. In our analysis we used the information on household income, parental educational level, parental working status, nationality, and single parenthood.

#### Household income

The household equivalent income is calculated by weighting the monthly net income according to the size and age composition of the household members. As weighting factors we used those of the 'OECD-modified scale' [19,20], which assigns a value of 1 to the household head, of 0.5 to each additional adult member, and of 0.3 to each child. Based on this variable a threshold for relative poverty is calculated as an income lower than the 60% of the regional median income of families as assessed in this survey (median = 1,309.52 EUR), and three income groups 'low' (< 60% of median, relative poverty), 'medium' (60% of median - median), and 'high' (> median) are differentiated.

An additional income group consists of those parents who did not indicate their income (i.e. 'missing values'). This additional group was created in order to avoid a possible bias due to the high number of parents who gave no information on their income (n = 503) [21]. Given, however, the high percentage of this group with missing income data (39.8%), we also explored whether this group shows differences from the group of parents that provided income data. Looking at the educational level, we found that in the group of parents with high educational level 66.9% indicated their income and 33.1% did not. A similar, but less pronounced difference was seen in the group with medium level education (59.3% vs. 40.7%). This difference all but disappeared in the low educational level group (50.4% vs. 49.6%). The association between missing income data and educational level was statistically significant (p-value < .0001). Looking at the nationality, we found that in the group with German nationality 62.6% indicated their income and 37.4 did not (p-value = 0.0126). No differences were found in parental working status and single parenthood at a 0.05 level of significance.

#### Parental educational level

This variable, divided in three categories, refers to the highest level of completed education reached by either the mother or the father. The highest level (i.e. 'high') refers to the completion of at least undergraduate studies or to a general qualification for university entrance but no completed study, the educational level labelled as 'medium' equals holding an upper secondary school certificate, a low educational level refers to a lower secondary school certificate or to not having completed any school. This categorisation yields a relatively high percentage of parents with a high educational level (54.6%), which, however, reflects the state of the situation in Munich, where the percentage of residents holding a high educational level or performing high qualified jobs is much higher than in other German cities [22].

#### Parental working status

Parental working status refers to *at least *one parent. A parent is considered 'not unemployed' either when he/she is full-or part-time employed or when he/she is not looking for a job. This implies that a parent not in labour force, such as a housewife or a student, is also considered 'not unemployed'. Unemployed are in our definition those who have explicitly stated this.

#### Nationality

Nationality is considered to be 'German' only if the child has *exclusively *the German citizenship. Double citizenship, similarly to not having the German citizenship, is categorized as 'non-German'.

#### Single parenthood

Single parenthood is determined by combining the answers relating to family status, living together with a partner, and being a single parent. The combination of the answers given to the respective question was necessary in order to minimize classification errors. For example, in a few cases, it was stated to be both a single parent and to live together with a partner. These answers were excluded from the classification as 'single parent'. Included were only those with consistent responses to all questions above.

### SDQ

The SDQ questionnaire, freely available on the Internet [9] in many different languages, consists of 25 items divided in 5 scales:

1) emotional symptoms scale

2) conduct problems scale

3) hyperactivity scale

4) peer problems scale

5) prosocial scale

For each scale the score can range from 0 to 10. With the exception of the prosocial scale, the scores from all the scales are added together to generate the SDQ-TDS. According to the scores, the SDQ-TDS is then classified as 'normal' (0-13), 'borderline' (14-16), and 'abnormal' (17-40). An abnormal score can then be used to identify likely 'cases' with mental health disorders [9].

### Statistical analysis

Prevalence estimates for the SDQ-TDS with 95% confidence interval (CI) were calculated. Chi square statistic along with its associated p-value was used to test whether the association between parental socioeconomic position and mental health problems is statistically significant. Crude and adjusted odds ratios (OR) with 95% CI for the SDQ-TDS as outcome variable were also calculated using logistic regression analysis. In order to select the variables in the logistic model a backward selection approach was used. The correlation between income and educational level was measured with Kendall's coefficient of rank correlation. A level of association ≥ |0.25| was considered to indicate a positive association. Since we obtained for these variables a Kendall's coefficient of 0.23 they were entered simultaneously in the regression. The statistical analysis was conducted with the software package SAS ^® ^version 9.1 (SAS Institute Inc., Cary, NC, USA).

## Results

The distribution of the social variables is given in table 1. It shows, *inter alia*, that about 11% of the boys and girls live in relative poverty with the regional median income of families within this survey as reference and that about 18% of the girls and 20% of the boys belong to families whose parents have a low educational level. The prevalence of mental health difficulties is presented in table 2. It shows that about 9% (CI 7.5 - 10.8) of the children has either an abnormal or a borderline 'total difficulties score', i.e. 10.5% (CI 8.2 - 13.2) of the boys and 6.3% (CI 4.5 - 8.5) of the girls.

**Table 1:** Basic distribution of the social variables

	**girls**		**boys**		**girls & boys**	
	
	**N**	**%**	**N**	**%**	**N**	**%**
**sex**	637	50.4	628	49.6	1265	100
**parental education**						
- high	363	57.0	328	52.2	691	54.6
- medium	140	22.0	157	25.0	297	23.5
- low	112	17.6	128	20.4	240	19.0
- missing value	22	3.5	15	2.4	37	2.9
**household income**						
- high	202	31.7	181	28.8	383	30.2
- medium	126	19.8	118	18.8	244	19.3
- low	68	10.7	67	10.7	135	10.7
- missing value	241	37.8	262	41.7	503	39.8
**parental working status**						
- not unemployed	541	84.9	529	84.2	1070	84.6
- unemployed	77	12.1	82	13.1	159	12.6
- missing value	19	3.0	17	2.7	36	2.8
**nationality**						
- (exclusively) German	471	73.9	440	70.1	911	72.0
- others	165	25.9	183	29.1	348	27.5
- missing value	1	0.16	5	0.8	6	0.5
**single parenthood**						
- others	547	85.9	524	83.4	1071	84.7
- single parent	84	13.2	97	15.5	181	14.3
- missing value	6	0.9	7	1.1	13	1.0

**Table 2:** Prevalence of normal, borderline and abnormal mental difficulties (based on the SDQ-Total Difficulties Score)

	**girls**		**boys**			**girls & boys**
	
	**N**	**%** **(95% Confidence Interval)**	**N**	**%** **(95% Confidence Interval)**	**N**	**%** **(95% Confidence Interval)**
SDQ-Total Difficulties Score						
0-13^a^	549	86.2 (83.3 - 88.8)	517	82.3 (79.1 - 85.2)	1066	91.0 (89.2 - 92.5)
14-16^b^	21	3.6 (2.2 - 5. 4)	32	5.5 (3.8 - 7.7)	53	4.5 (3.4 - 5.9)
17-40^c^	19	3.2 (2.0 - 5.0)	34	5.8 (4.1 - 8.0)	53	4.5 (3.4 - 5.9)
≥ 14^d^	40	6.3 (4.5 - 8.5)	66	10.5 (8.2 - 13.2)	106	9.0 (7.5 - 10.8)

a) normal

b) borderline

c) abnormal

d) borderline or abnormal

The prevalence of mental health difficulties is by far the lowest when the educational level or the household income of the parents is the highest. The corresponding figures for the low and the high educational group are 18.4% vs. 8.4% for the boys and 9.7% vs. 3.6% for the girls (table 3). The p-value is 0.0141 for the boys and 0.0038 for the girls, indicating that these associations are statistically significant. For the low and high income groups the corresponding figures are 13.6 vs. 6.3 for the boys and 14.3 vs. 4.7 for the girls. The p-value is 0.0147 when the analysis is made for girls and boys together, and above the significance level of 0.05 when a separate analysis is performed (0.0715 for the girls and 0.0873 for the boys). The prevalence of mental health difficulties is quite similar in the medium and low educational groups (11.6 vs. 14.3) and in the medium and low income group (10.8 vs. 13.9), and these prevalances are much higher than in the most advantaged education and income groups (5.9 and 5.4, respectively).

**Table 3:** Socioeconomic differences in the prevalence of borderline or abnormal mental difficulties (based on the SDQ-Total Difficulties Score)

	**mental difficulties**					
	**girls**		**boys**		**girls & boys**	
	
	**N**	**%**	**N**	**%**	**N**	**%**
**parental education**						
- high	12	3.6	26	8.4	38	5.9
- medium	15	11.1	18	12.1	33	11.6
- low	10	9.7	21	18.4	31	14.3
p-value^a^	0.0038		0.0141		0.0002	
**household income**						
- high	9	4.7	11	6.3	20	5.4
- medium	8	6.9	17	14.7	25	10.8
- low	9	14.3	8	13.6	17	13.9
- not indicated	14	6.5	30	12.9	44	9.8
p-value^a^	0.0715		0.0873		0.0147	
**parental working status**						
- not unemployed	30	5.9	51	10.3	81	8.0
- unemployed	7	10.1	12	16.0	19	13.2
p-value^a^	0.1774		0.1407		0.0413	
**nationality**						
- (exclusively) German	27	6.1	42	10.0	69	8.0
- others	13	9.2	24	15.1	37	12.3
p-value^a^	0.2012		0.0852		0.0245	
**single parenthood**						
- others	28	5.5	53	10.9	81	8.1
- single parent	12	15.6	13	14.1	25	14.8
p-value^a^	0.0011		0.3650		0.0053	

a) chi^2^-Test

A similar, but less pronounced association is also seen for parental working status, nationality, and single parenthood, always indicating that social disadvantage is associated with higher prevalence of mental difficulties.

The logistic regression models also give evidence that parental education and household income are the strongest independent variables associated with mental difficulties (table 4). The crude OR for the outcome variable SDQ-TDS shows that boys and girls whose parents have the lowest educational level and the lowest household income are approximately three times more likely to have mental difficulties compared to children whose parents have the highest educational level. The corresponding figures are 2.7 (CI 1.6 - 4.4) for the low educational level and 2.8 (CI 1.4 - 5.6) for the low household income. While adjustment for the other socioeconomic indicators (table 5) accounted for limited changes of the OR relating to low parental education (OR = 2; CI 1.2 - 3.5), it reduced notably the independent effect of low household income (OR = 1.7 CI 0.8 - 3.6). Adjusted odds ratios for both girls and boys also show that a medium parental education and a medium household income have a similar impact on the mental difficulties of children as a low parental education and low household income (medium vs. low parental education = 1.9 vs. 2.0; medium vs. low parental household income = 1.6 vs. 1.7).

**Table 4:** Unadjusted Odds Ratios for borderline or abnormal mental difficulties (based on the SDQ-Total Difficulties Score)

	**Odds Ratios for mental difficulties (95% Confidence Interval)**		
	**girls**	**boys**	**girls & boys**
	
**parental education**			
- high^a^	1.0	1.0	1.0
- medium	3.4 (1.5 - 7.4)	1.5 (0.8 - 2.8)	2.1 (1.3 - 3.4)
- low	2.9 (1.2 - 6.9)	2.5 (1.3 - 4.6)	2.7 (1.6 - 4.4)
**household income**			
- high^a^	1.0	1.0	1.0
- medium	1.5 (0.6 - 4.0)	2.6 (1.2 - 5.7)	2.1 (1.1 - 3.9)
- low	3.4 (1.3 - 9.0)	2.3 (0.9 - 6.1)	2.8 (1.4 - 5.6)
- not indicated	1.4 (0.6 - 3.3)	2.2 (1.1 - 4.5)	1.9 (1.1 - 3.3)
**parental working status**			
- not unemployed^a^	1.0	1.0	1.0
- unemployed	1.8 (0.9 - 4.3)	1.7 (0.8 - 3.3)	1.7 (1.0 - 3.0)
**nationality**			
- (exclusively) German^a^	1.0	1.0	1.0
- others	1.6 (0.8 - 3.1)	1.6 (0.9 - 2.7)	1.6 (1.0 - 2.5)
**single parenthood**			
- others^a^	1.0	1.0	1.0
- single parent	3.2 (1.5 - 6.5)	1.4 (0.7 - 2.6)	2.0 (1.2 - 3.2)

a) reference

**Table 5:** Adjusted Odds Ratios for borderline or abnormal mental difficulties (based on the SDQ-Total Difficulties Score)

	**Odds Ratios for mental difficulties (95% Confidence Interval)**		
	**girls**	**boys**	**girls & boys**
	
**parental education^b^**			
- high^a^	1.0	1.0	1.0
- middle	3.1 (1.4 - 7.0)	1.3 (0.7 - 2.5)	1.9 (1.1 - 3.1)
- low	2.3 (0.9 - 6.1)	1.9 (1.0 - 3.7)	2.0 (1.2 - 3.5)
**household income^c^**			
- high^a^	1.0	1.0	1.0
- middle	1.0 (0.4 - 2.9)	2.2 (0.9 - 5.1)	1.6 (0.8 - 3.1)
- low	1.7 (0.5 - 5.4)	1.7 (0.6 - 4.9)	1.7 (0.8 - 3.6)
- not indicated	0.9 (0.3 - 2.3)	1.9 (0.8 - 4.1)	1.4 (0.8 - 2.5)
**parental working status^d^**			
- not unemployed^a^	1.0	1.0	1.0
- unemployed	1.0 (0.4 - 2.5)	1.4 (0.7 - 2.9)	1.2 (0.7 - 2.1)
**nationality^e^**			
- (exclusively) German^a^	1.0	1.0	1.0
- others	1.4 (0.7 - 3.0)	1.5 (0.8 - 2.6)	1.5 (0.9 - 2.4)
**single parenthood^f^**			
- others^a^	1.0	1.0	1.0
- single parent	2.2 (0.9 - 5.0)	1.2 (0.6 - 2.5)	1.6 (0.9 - 2.8)

a) reference

b) adjusted for household income, parental working status, nationality, and single

parenthood

c) adjusted for parental education, parental working status, nationality, and single parenthood

d) adjusted for parental education, household income, nationality, and single parenthood

e) adjusted for parental education, household income, parental working status, and single parenthood

f) adjusted for parental education, household income, parental working status, and nationality

By performing a selection of the variables with a backward selection (analysis not presented here in tables) the effect of parental education (low vs. high) remains relatively stable for both boys and girls (OR = 2.6; CI 1.4 - 4.9 and OR = 2.7; CI = 1.1 - 6.5, respectively). Instead, the independent effect of household income disappears.

In addition to parental education and household income, single parenthood seems to be associated significantly with the psychological health of the children. Children of single parents have, in fact, a higher probability (OR = 2.0; CI 1.2 - 3.2) of a borderline or abnormal SDQ-TDS (as compared to those children who live with both parents). By performing, however, a separate logistic regression analysis for boys and girls with backward selection (analysis not presented here in tables), the independent effect of single parenthood disappears for the boys when other socioeconomic variables (i.e. parental education, household income, parental working status, and nationality) are controlled for. The effect only remains statistically significant for the girls, though (OR = 2.4; CI 1.1 - 5.3).

In order to assess the coverage of children mental health specialists in Munich, and to be able to compare them with the available data for the national level, which are based on rates for the population 14 years and younger [15], we first calculated the rate of mental health specialists in Munich for the population group '14 years or younger'. As we did not have the exact number of children less than 15 years old living in Munich, but only the percentage of young people under 20 years (i.e. 16.9%) [18], we subtracted from it one quartile. This calculation is based on the realistic assumption that the Munich population under 20 years is relatively stable across the quartiles. The resulting figure (about 171,450 children; i.e. 12.7% of the total population) was compared with the number of children mental health specialists in Munich. Working with these data, we found that in Munich the rate of *child psychologists *per 100,000 children below 15 years is 75.2, and that the rate of *child psychiatrists *is 8.2. If, following the WHO [23], one also includes the number of specialists in psychosomatics and psychotherapy in the rate of *psychiatrists for adults and children *per 100,000 persons, the overall rate of psychiatrists is 30.6 for Munich.

Comparing these rates with those available at the national level we found that the rate of children mental health specialists is in Munich much higher than in Germany as a whole. In Munich, in fact, the rate of *child psychologist *per 100,000 (rate for the population below age 15) is more than 5 times higher than in the rest of the country (75/100,000 vs. 14/100,000, respectively) and it is comparable to the highest rates in Europe such as those of Belgium (82/100,000) and Switzerland (81/100,000) [15]. The Munich rate of *child psychiatrists *equals approximately the national rate (8/100,000 vs. 9/100,000). If one also includes specialists in psychosomatics and psychotherapy, the overall rate of *psychiatrists for adults and children *per 100,000 persons is almost three times higher in Munich as compared with the national rate (31/100,000 vs. 12/100,000). It can be speculated that the rate of child psychiatrists is much higher for Munich than for Germany as whole, if child specialists in psychosomatics and psychotherapy were also included (but these data were not available).

## Discussion

Our results indicate that childhood mental difficulties follow a socioeconomic pattern in Munich as well, and that this pattern is similar to the one seen in the German-wide KiGGS study mentioned in the introduction. There is an important difference between the results from Munich and Germany, though: the prevalence of children who have a borderline or abnormal SDQ-TDS is about 30% lower in Munich than in Germany as a whole, as reported by the KiGGS for the age group 3-6 (9.0% vs. 13.2%). The prevalence of a borderline or abnormal SDQ-TDS among boys is, like in the KiGGS study, notably higher than among girls (10.5% vs. 6.3% in our analysis and 15.8 vs. 10.6 in the KiGGS study, respectively). The statement that the prevalence of mental difficulties is considerably lower in Munich than in Germany is probably not affected by the fact that the age of our Munich sample (median = 5.9) does not completely match the age of the KiGGS comparison group (3-6 years), as in the KiGGS study the children aged 3-6 (along with the adolescents aged 14-17) show the lowest proportion of mental health difficulties.

At the international level, the prevalence estimates of mental health problems vary enormously. A study investigating the epidemiology of childhood and adolescent psychiatric disorders found that the median prevalence estimate is 12% across different studies [24]. This would imply that the prevalence estimate we found for Munich (i.e. 9.0%) is not only lower than the German national level, but also lower than that reported in most other studies.

If living in a city with an outstanding quality of life and high availability of mental health specialists might offer some protection from developing mental health disorders in early age, this does not seem to protect the more disadvantaged children from being more affected than those who belong to a higher socioeconomic position. The distribution of mental health difficulties shows in fact a clear social gradient, as mental health difficulties among girls and boys progressively increase as the educational level or the household income of the parents decrease. Our findings show, in particular, that the parental education is the strongest risk factor for mental difficulties for both girls and boys (i.e. stronger than household income, parental working status, nationality or single parenthood). Similarly, at the national level, the KiGGS study showed that the parental education has an important impact on the health of the children. In particular, in the group of girls aged 11-17, the parental education has been identified as the strongest risk factor for mental health difficulties [25]. In addition to parental education, we also found that single parenthood is positively associated with a borderline/abnormal SDQ-TDS for the girls. This might be explained with the hypothesis that girls are more vulnerable to psychosocial stressors than boys, and that there are gender differences in stress responses [26].

In the KiGGS analysis, the results of the logistic regression show that family structure (single parenthood/youth institution) and migration (at least one parent not born in Germany and children immigrated or both parents not having the German citizenship or both parents immigrated) are risk factors for both girls and boys. These results, however, refer to the total KiGGS sample aged 3-17 years; as such they are not well suited to be compared with our sample of children aged 5-7 years. It might be speculated that with a larger sample these variables could have been significant for Munich as well, but our results seem rather to indicate that when parental education is controlled for, the other social variables such as household income, parental working status, nationality, and single parenthood no longer have a significant impact on the mental health of the children.

Our results show that socioeconomic disparities in mental health persist even in a place that, in addition to a high quality of life, also has a particular good coverage of and access to mental health services. In Munich, in fact, like in the whole country, access to health services is almost universal. These findings make it hard to argue that the origins of mental health disparities lie in a possible gap concerning supply with mental health specialists. It can be hypothesized that barriers such as lack of awareness and acceptance of mental health problems are especially high in low status groups, but as far as we know there is no study testing this hypothesis in more detail. In our study as well we could not assess and quantify these barriers. Also, the question whether children with similar mental difficulties make the same use of mental health services, *independently of *their socioeconomic position, could not be addressed here. Our study indicates, though, that a very good coverage of mental health specialists *per se *does not have a significant effect on the social distribution of mental difficulties among children. Rather, this suggests a possible failure of the German health care system in detecting cases in need of better care at an early stage (i.e. before children enter the school system) and, more specifically, a failure in employing mental health specialists in the prevention of mental difficulties among high-risk groups.

The socioeconomic position of the parents, and in particular the parental education, seems to play the most important role for a healthy mental development of their children, much more important than the region where children live or the availability of mental health care services. This finding supports the argument that those stressful influences to which children living in disadvantaged educational groups are exposed form the basis of mental health disparities [27]. Living in a family with low educational background seems to be a risk factor per se, while the fact that children live in a prosperous city and the availability of mental health services has an effect on the overall prevalence, but not on the socioeconomic pattern. In other words, a favourable *external environment*, as opposed to the *family environment*, does not seem to be able to fight the roots of health disparities.

The main question is how to move from the description of socioeconomic differences in mental health to the exploration of strategies to reduce those disparities. If, in fact, even in a city such as Munich the socioeconomic pattern reflects that of the national level, it can be hypothesized that the same disparities can also be found in other regions. The overall prevalence will change from region to region, but the socioeconomic pattern will probably always be the same, pointing to the fundamental problem that these disparities are very stable and that they can hardly be changed by single, short-term interventions.

Phrased in very general terms, public health programs should be developed in order to have an impact especially on those family environments where the most disadvantaged children live [28]. In the USA an evidence-based program, the Nurse-Family Partnership, aimed at improving the health and self-sufficiency of lower socioeconomic families, has proven to be successful in addressing the roots of health disparities among children. Randomised controlled trials conducted over three decades showed that the program is cost-effective and able, for example, to increase maternal employment and school readiness of children born to mothers with low psychological resources. Among the benefits there is also a 67% reduction in behavioural and intellectual problems among children [29]. In Germany, although there are some promising projects along this way [30], a rigorous evaluation of such programs is still lacking as the social epidemiological research is more focused on measuring and exploring different aspects of health inequalities than on implementing and evaluating public health interventions aimed at reducing these gaps. The building and the evaluation of such programs remains an important area for social epidemiological research in the near future in Germany and in other countries where this is not yet established.

Concerning potential problems of our dataset and analyses, they are mostly to be attributed to the relatively small sample size. This has probably prevented us from identifying other important risk factors such as, for example, parental unemployment. Another limitation of our study lies in the many missing values of the variable 'household income'. We believe, however, that our results are to be trusted, as the response rate is rather high (i.e. 69%), and as the SDQ is a well-established instrument.

## Conclusions

The distribution of mental health difficulties among children has a pronounced socioeconomic pattern, with prevalences especially high for children from parents with low educational level. This gap can be seen in Germany as a whole and also in a city such as Munich with its comparatively high standard of living and with a number of mental health specialists that is far higher than the national average and among the highest in Europe. This study strengthens the hypothesis that the roots of mental health disparities among children can mostly be found in the family environment, as opposed to the regional environment, and that the regional context could have a strong impact on the overall prevalence, but not on the social disparities in mental health. In Germany, there is a need for social epidemiology to shift focus away from the description of socioeconomic disparities in mental health towards intervention and evaluation of programs directed at reducing those disparities.

## Competing interests

The authors declare that they have no competing interests.

## Authors' contributions

LP wrote the paper, performed the statistical analysis, and designed the study. GB designed and coordinated the GME survey, provided insights on the dataset, contributed to the writing, and commented on drafts. HM and GS commented on drafts. AM participated in the design of the study, contributed to the writing and commented on drafts. All authors read and approved the final manuscript.

## Pre-publication history

The pre-publication history for this paper can be accessed here:

http://www.biomedcentral.com/1471-2458/10/199/prepub
